# Does treating obesity stabilize chronic kidney disease?

**DOI:** 10.1186/1471-2369-6-7

**Published:** 2005-06-15

**Authors:** Sujata Agnani, Vidula T Vachharajani, Rohit Gupta, Naveen K Atray, Tushar J Vachharajani

**Affiliations:** 1 Department of Medicine and Nephrology, Louisiana State University Health Sciences Center, Shreveport, Louisiana, USA; 2 Department of Nephrology, Overton Brooks Veterans Affairs Medical Center, Shreveport, Louisiana, USA

## Abstract

**Background:**

Obesity is a growing health issue in the Western world. Obesity, as part of the metabolic syndrome adds to the morbidity and mortality. The incidence of diabetes and hypertension, two primary etiological factors for chronic renal failure, is significantly higher with obesity. We report a case with morbid obesity whose renal function was stabilized with aggressive management of his obesity.

**Case report:**

A 43-year old morbidly obese Caucasian male was referred for evaluation of his chronic renal failure. He had been hypertensive with well controlled blood pressure with a body mass index of 46 and a baseline serum creatinine of 4.3 mg/dl (estimated glomerular filtration rate of 16 ml/min). He had failed all conservative attempts at weight reduction and hence was referred for a gastric by-pass surgery. Following the bariatric surgery he had approximately 90 lbs. weight loss over 8-months and his serum creatinine stabilized to 4.0 mg/dl.

**Conclusion:**

Obesity appears to be an independent risk factor for renal failure. Targeting obesity is beneficial not only for better control of hypertension and diabetes, but also possibly helps stabilization of chronic kidney failure.

## Background

Obesity is a major health problem in the western world. Nearly two-thirds of the U.S. adults are overweight (BMI > 25) and of these one-half are obese (BMI >30) [1]. Obesity is not only associated with increase in morbidity, mortality and reduction in life expectancy [2], but also leads to increase in the incidence of diabetes [3], hypertension [4], dyslipidemia and coronary artery disease [5]. Both diabetes and hypertension together account for approximately 70% of end-stage renal disease (ESRD).

Approximately 300,000 adult deaths in the United States each year are attributable to unhealthy dietary habits and physical inactivity or sedentary behavior, with obese individuals having a 50 to 100 percent increased risk of death from all causes; most of the increased risk is due to cardiovascular causes [6,7]. Obesity has also resulted in an increase in the cluster of disorders often referred to as the "metabolic syndrome". Although kidney disease has not yet been recognized as a major component of this metabolic syndrome, accumulating evidence suggests that even in non-diabetic obese patients, there is some degree of renal dysfunction that can lead to more serious injury to the kidneys as metabolic and hemodynamic disturbances worsen with prolonged obesity [8,9].

We report a case that illustrates the stabilization of renal function with obesity directed therapy.

## Case Report

A 43-year-old Caucasian male was referred to the nephrology clinic at Overton Brooks VAMC by his primary care practitioner, in November of 2002 for management of his chronic kidney disease. He was asymptomatic. His BP was well controlled at 115/83 mmHg. He was morbidly obese with a body mass index (BMI) of 46, chronic kidney disease stage 4 (MDRD GFR of 16 ml/min), non insulin dependant diabetes mellitus, hypertension, coronary artery disease status post stent placement and hyperlipidemia. His medications included nifedipine, fosinopril, atenolol, rosiglitazone, furosemide, simvastatin, aspirin, glyburide and calcium carbonate. Laboratory results: Serum creatinine 4.3 mg/dl, BUN 54 mg/dl, normal electrolytes, serum calcium 8.8 mg/dl, serum phosphorus 4.9 mg/dl, random urine protein 292 mg/dl, random urine creatinine 49 mg/dl, urine protein/creatinine ratio of 5.9, hemoglobin A1c 7% and hemoglobin 13.9 g/dl. Patient was educated about the course and prognosis of his kidney disease and advised diet and exercise for weight loss. He was referred for arterial-venous fistula placement for providing renal replacement therapy in future. Over the next 6 months the patient failed all conservative methods of weight loss including the use of orlistat. His morbid obesity posed a major contraindication for enrolling him for kidney transplantation. He agreed to the surgical therapy option for treating his obesity. He was referred for bariatric surgery in June 2003.

**Figure 1:**
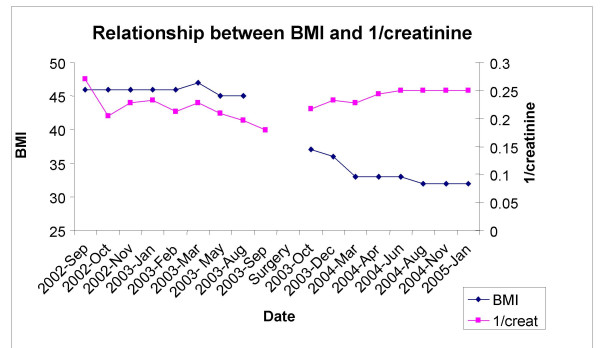
Relationship between renal function as 1/creatinine and BMI.

After the bariatric surgery in September 2003, he had lost 60 pounds at 6 months (BMI 37). He was able to discontinue all his oral hypoglycemic agents maintaining a hemoglobin A1c of 6.2% and required only one anti-hypertensive medication to achieve the recommended target blood pressure reading. His BUN and creatinine has remained at 22 mg/dl and 4.6 mg/dl respectively. The patient is being followed at regular intervals and over the course of the next eight months has lost an additional 30 pounds (BMI 32), a total weight loss of 90 pounds since the bariatric surgery. His serum creatinine has remained stable at 4 mg/dl, BUN 37 mg/dl, random urinary protein 99 mg/dl, random urinary creatinine 121 mg/dl, urine protein/creatinine ratio of 0.8 and hemoglobin A1c 5.1%. The inverse creatinine to time plot as shown in figure 1 clearly demonstrates the stabilization of the renal function 15 months following his weight loss surgery. The patient was being evaluated for pre-emptive renal transplantation and because of his previous history of coronary artery disease he underwent a left heart catheterization study in March 2005. Unfortunately, despite all precautions he developed radio-contrast induced nephropathy and had to be initiated on renal replacement therapy. He currently remains on dialysis and is awaiting a renal transplantation, which would not have been possible without his weight loss.

## Discussion

The case report presented here illustrates the benefits of weight reduction on the progression of kidney disease. There are few studies investigating the pathophysiology of obesity and its early effects on kidney structure and function. Clinical as well as laboratory animal studies have suggested the role of glomerular hypertension due to renal vasodilatation and increase in hydrostatic pressure leading to increased glomerular wall stress and increased tubular sodium absorption [10,11]. The other proposed mechanism of excessive tubular sodium re-absorption include increased intra-renal pressures caused by the excess accumulation of adipose tissue in the viscera with compression of the loop of Henle and vasa recta leading to sluggish flow in the renal tubules and vasa recta and thus causing an increase in the tubular sodium re-absorption [9,12]. Increased sodium re-absorption in the loop of Henle initially reduces the macula densa sodium chloride delivery thereby initiating a macula densa feedback that causes vasodilatation of afferent arterioles; this increases renin secretion despite sodium retention and volume expansion. The compensatory vasodilatation of afferent arterioles resulting in an initial rise in glomerular filtration rate (GFR) and increase in the glomerular wall stress leads to increased extra cellular matrix formation and fibrosis along with injury to the kidneys and nephron loss with a resultant decrease in GFR over a prolonged period of time.

There is also a growing body of evidence that obesity per se is a pro inflammatory state. Obesity is associated with increased levels of acute phase reactants, and cytokines as well as reactive oxygen species [13,14]. Glomerular hyperfiltration also causes loss of protein in urine, which promotes glomerular inflammatory responses thus leading to progression of chronic kidney disease. In the already pre-existing pro-inflammatory state of obesity this could have an additive effect.

Proteinuria seen in obese patients is often considered to be secondary to focal and segmental glomerulosclerosis. However, Kambham et al have reported a distinct obesity related histopathological change in the glomeruli, referred to as obesity-related glomerulopathy and was characterized by glomerulomegaly and focal segmental glomerulosclerosis. This entity defers from idiopathic focal segmental sclerosis with a lower incidence of nephrotic syndrome, more indolent course, consistent presence of glomerulomegaly, and milder foot process fusion [15].

We did not perform a renal biopsy hence we do not know whether proteinuria was secondary to obesity related glomerulopathy or idiopathic focal segmental sclerosis. Adequate treatment of obesity reduces proteinuria and decreases the need for medications such as angiotensin converting enzyme inhibitors or angiotensin-receptor blockers, which are known to further reduce the glomerular filtration rate.

Hence targeting obesity should benefit in maintaining and preserving kidney function, regardless of whether weight reduction is achieved by diet and exercise or by bariatric surgery. Even though there have been no large studies directly comparing the effectiveness of different methods of weight loss on progression of kidney dysfunction, there is little doubt that weight loss reduces hypertension and type 2 diabetes, the two main risk factors for development of end stage renal disease. Bariatric surgery is a rapidly evolving branch of surgical science with the aim to induce major weight loss in those whose obesity places them at high risk of serious health problems. Bariatric surgery leads to withdrawal of anti-diabetic therapy in about 60% of patients, and reduction of therapy for many other diabetic patients. Eighty-two percent of the 165 type-2 diabetes mellitus patients in the uncontrolled observational study by Greenville achieved target glycemic control without any medications after an average of 14-years follow up [16]. The SOS study was a well-designed prospective study on obese patients in which, bariatric surgery was compared with a non-surgical group. The glycemic control without medication after 2-years in the surgical group was achieved in twice the number of patients compared to the control group [17].

Alexander et al studied 30 morbidly obese patients; 19 with chronic kidney disease and 11 with renal transplantation; and reported gastric bypass surgery to be an effective means for achieving significant long-term weight loss and relief of co-morbid conditions in patients with renal failure on dialysis, in preparation for transplantation, or after transplantation [18].

The above observations that bariatric surgery leads to reduction in the risk factors associated with development of ESRD and our case report showing that the progression of CKD was retarded with postponement of dialysis raises the question: Should bariatric surgery be recommended in the morbidly obese who fail to achieve sufficient weight loss using non-surgical approaches especially those who are young and have other metabolic syndrome risk factors and are at a favorable anesthetic/surgical risk?

## Competing interests

The author(s) declare that they have no competing interests.

## Authors' contributions

SA: Collected the data and drafted the manuscript; VTV: Participated in the coordination and drafting of manuscript; RG: participated in the data collection and formatting of the manuscript NKA: participated in the clinical management of the case and drafting and finalizing the manuscript and TJV: Conceived the idea, coordinated the data and helped in drafting and finalizing the manuscript. All authors have read and approved the manuscript.

## Pre-publication history

The pre-publication history for this paper can be accessed here:
